# Supplementary material for: Correction: Serum soluble B7-H4 is a prognostic marker for patients with non-metastatic clear cell renal cell carcinoma
Source: PLoS One. 2018 Aug 23;13(8):e0203174. doi: 10.1371/journal.pone.0203174 (PMC6107280; doi:10.1371/journal.pone.0203174)
Supplement: S1 File — (PDF) [file pone.0203174.s001.pdf]

PLOS ONE

RESEARCH ARTICLE

Serum soluble B7-H4 is a prognostic marker for patients with non-metastatic clear cell renal cell carcinoma

Takeshi Azuma[1,2,3]*, Yujiro Sato[1], Tetsukuni Ohno[2], Miyuki Azuma[2], Haruki Kume[3]

1 Department of Urology, Tokyo Metropolitan Tama Medical Center, Tokyo, Japan, **2** Department of Molecular Immunology, Graduate School of Medical and Dental Sciences, Tokyo Medical and Dental University, Tokyo, Japan, **3** Department of Urology, The University of Tokyo Graduate School of Medicine, Tokyo, Japan

* tazuma-tky@umin.ac.jp

Abstract

Background

B7-H4 is a member of the B7 family of immune-regulatory ligands and is considered to be a negative regulator of the immune response. We investigated the clinical significance of serum soluble B7-H4 in patients with non-metastatic clear cell renal cell carcinoma.

Methods

We analyzed 108 patients in whom non-metastatic clear cell renal cancer was diagnosed at Tokyo Metropolitan Tama Medical Center between 2008 and 2013. We measured the serum soluble B7-H4 level using the Enzyme-Linked ImmunoSorbent Assay (ELISA) and evaluated the association between the peripheral blood neutrophil count and sB7-H4 as well as the utility of soluble B7-H4 as a prognostic biomarker for clear cell renal cancer. The Cox proportional hazards regression model was used to assess the PFS and OS with the soluble B7-H4 level.

Results

We detected high levels of soluble B7-H4 in the sera of 56% of patients with non-metastatic clear cell renal cell carcinoma versus only 10% of healthy donors. Elevated soluble B7-H4 levels were associated with changes in an elevated peripheral blood neutrophil count. The increase of soluble B7-H4 also was significantly associated with poor PFS and OS. Multivariate analysis showed that the elevation of the soluble B7-H4 level was an independent prognostic factor for PFS and OS.

Conclusions

Our data suggest that the association between serum soluble B7-H4 and peripheral blood neutrophil count, as well as the evaluation of serum soluble B7-H4 expression is a useful tool for predicting the prognosis of patients with non-metastatic clear cell renal cell carcinoma.

Check for updates

OPEN ACCESS

Citation: Azuma T, Sato Y, Ohno T, Azuma M, Kume H (2018) Serum soluble B7-H4 is a prognostic marker for patients with non-metastatic clear cell renal cell carcinoma. PLoS ONE 13(7): e0199719. https://doi.org/10.1371/journal.pone.0199719

Editor: Aamir Ahmad, University of South Alabama Mitchell Cancer Institute, UNITED STATES

Received: May 2, 2018

Accepted: June 12, 2018

Published: July 25, 2018

Copyright: © 2018 Azuma et al. This is an open access article distributed under the terms of the Creative Commons Attribution License, which permits unrestricted use, distribution, and reproduction in any medium, provided the original author and source are credited.

Data Availability Statement: ***DATA PA AT ACCEPT*** Please note the following Manuscript Note: "NJS(EO)/12Jun: ***AT NEXT DECISION: author has data set which needs to be attached if still needed case #05805605" All relevant data are within the paper.

Funding: This study was supported by JSPS KAKENHI, Grant Number 16K11037 to Y. Sato and JSPS KAKENHI, Grant Number 17K11169 to T. Azuma. The funder had no role in study design,

data collection and analysis, decision to publish, or preparation of the manuscript.

Competing interests: The authors have declared that no competing interests exist.

Introduction

B7-H4 is a recently identified member of the B7 family of immune regulatory ligands and is considered to be a negative regulator of the immune response[1]. B7-H4 is reportedly expressed at high levels in many cancer tissues such as those of the breast, ovaries, lung, pancreas, renal cells, and stomach[2–6]. Some studies showed that it is associated with the development of several types of tumor. Functionally, B7-H4 can inhibit CD4 and CD8 T cell proliferation and cytokine production through unidentified B7-H4 receptors on T cells[7]. Interestingly, B7-H4 has also been shown to regulate neutrophil-mediated innate immune responses negatively[8]. The B7-H4 molecule has a soluble form in addition to the membrane-bound form. We previously reported that soluble B7-H4 acts as a decoy molecule to block the inhibitory functions of cell-surface B7-H4[9].

In this study, we investigated the association between serum soluble B7-H4 and the peripheral blood neutrophil count, as well as whether the presence of soluble B7-H4 was associated with poor a prognosis in non-metastatic clear cell renal cell carcinoma patients.

Materials and methods

Patients and healthy donors

Patients with non-metastatic clear cell renal cell carcinoma who were treated surgically at Tokyo Metropolitan Tama Medical Center between 2008 and 2013 and provided a preoperative serum sample were enrolled. Serum samples was preserved in -80˚C freezer. TNM stage was determined by the 2010 AJCC (American Joint Committee on Cancer) TNM classification. Healthy donors (HDs) with no history of cancer were recruited in the same period. The study and protocol were approved by the Ethical Review Board of Tama Medical Center and all the patients and HDs gave written informed consent for their participation in this study. The median follow-up period was 61.5 months.

Detection of soluble B7-H4

The sandwich Enzyme-Linked ImmunoSorbent Assay (ELISA) (LifeSpan BioScience, Seattle, United States) was used for the detection of human soluble B7-H4 (human VTCN1/B7-H4). Briefly, 25 μL of an undiluted blood specimen was added to high-binding polystyrene plates coated with capture antibody. Immobilized antigens were detected with a diluted biotinylated detection antibody followed by horseradish peroxidase-conjugated streptavidin. For calibration, the standards of recombinant protein and two controls were conducted in parallel with the test samples on each plate. Each experiment was performed in triplicate.

Statistical analysis

We performed statistical analysis with the Mann-Whitney U test for a single comparison of the parametric data. Correlations were analyzed using the Spearman rank test.

The distributions of the progression-free survival (PFS) rate and overall survival (OS) rate were determined using the Kaplan-Meier method. The relationship between survival and each parameter was analyzed using the log-rank test and the Cox regression model and summarized with the risk ratios and 95% confidence intervals (CI). The survival data were updated in January 2018. Statistical analyses were performed using the JMP® software package, and $p<0.05$ was considered statistically significant.

Results

The characteristics of renal cancer patients, including age, gender, the soluble B7-H4 level, and preoperative peripheral blood neutrophil count are summarized in Table 1. Serum samples were obtained from 108 patients with diagnosed renal cancer (69 men and 39 women, mean age = 65.1 y; age range: 32–88 y) and 108 HDs (69 men and 39 women, mean age = 64.5 y; age range: 42–85 y) as controls.

To detect soluble B7-H4, sera from individual patients with a diagnosis of renal cancer were analyzed using ELISA. In this assay, 56% (60 out of 108) of the samples from patients with renal cancer were above the background for soluble B7-H4 and therefore considered positive. Assessment of soluble B7-H4 in the HDs showed that only 10% (11 out of 108) were positive ($P < 0.001$) (Fig 1). The levels of soluble B7-H4 were also significantly higher in renal cancer patients than in the HDs; the mean concentration of soluble B7-H4 in renal cancer patients (167.1 ng/mL in all renal cancer patients and 295.1 ng/ml in positive renal cancer patients with a range of 0.11 to 1021 ng/mL) was significantly higher than in the HDs (0.9 ng/mL in all HDs and 8.5 ng/ml in positive HDs with a range of 0 to 21 ng/mL).

In our previous study we reported an association between soluble B7-H4 and neutrophils in an infection and arthritis model[8, 9]. In our assessment we found a significant between serum soluble B7-H4 and the preoperative peripheral blood neutrophil count, one of the prognostic markers in renal cancer patients ($y = 0.07x–19.6$, $R^2 = 0.428$, $T<0.05$, Fig 2).

The serum soluble B7-H4-positive and negative groups differed significantly in terms of their PFS and OS (Fig 3). The PFS rate for the serum soluble B7-H4-positive and negative groups at 5 years was 91.3 and 95.6%, respectively. The OS rate for the serum soluble B7-H4-positive and negative groups at 5 years was 65.4 and 75.0%, respectively. Univariate Cox regression showed that the serum soluble B7-H4-positive group was significantly associated with a poor clinical outcome (Tables 2 and 3). The previously reported factors, including the pathological T stage, grade, and preoperative peripheral neutrophil count, also correlated with the PFS and OS (Tables 2 and 3).

The multivariate Cox proportional hazards regression model including age, gender, pathological T stage, grade, preoperative peripheral neutrophil count, and serum soluble B7-H4 revealed that the presence of serum soluble B7-H4 was an independent predictive marker of PFS and OS (Tables 2 and 3).

Table 1. Patient characteristics.

		Serum soluble B7-H4 level		
		Negative (n = 48)	Positive (n = 60)	p value
**Age (years ± SD)**		65.3±11.9	64.9±12.5	0.873
	>65	24	33	0.605
	≤65	24	27	
**Gender**	Male	31	38	0.893
	Female	17	22	
**Grade**	G1-2	47	49	0.008
	G3-4	1	11	
**T stage**	T1-2	39 (23%)	51 (31%)	0.603
	T3-4	9 (20%)	9 (26%)	
**Preoperative neutrophil count**	≤6000	48 (37%)	51 (37%)	0.005
	>6000	0 (6%)	9 (20%)	

https://doi.org/10.1371/journal.pone.0199719.t001

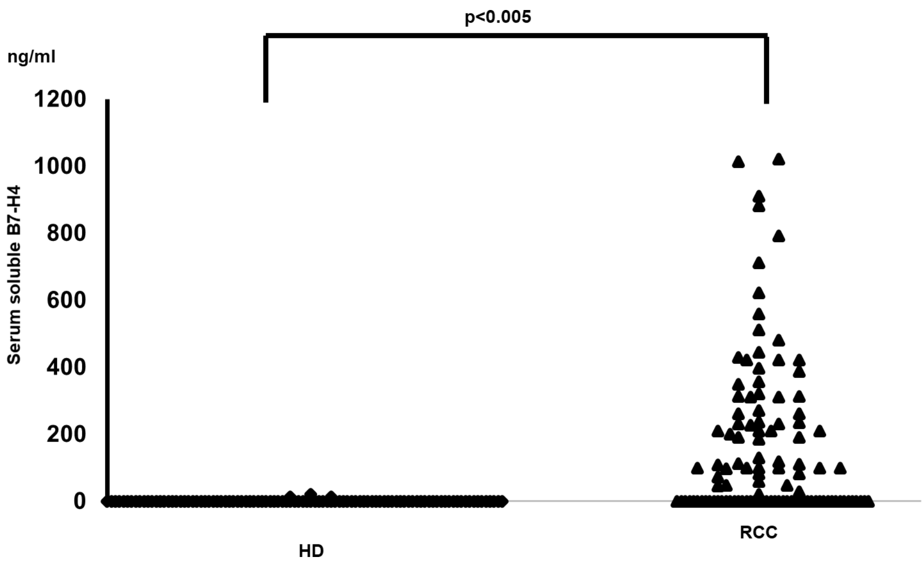

Fig 1. Detection of serum soluble B7-H4 in patients with non-metastatic clear cell renal cell carcinoma. The concentration of the soluble B7-H4 in the HDs and the patients diagnosed with renal cell carcinoma. Sera from 108 renal cancer patients and 108 HDs were diluted 1:10 in PBS and tested by ELISA as described in Materials and Methods. The data were analyzed using the Mann-Whitney U test followed by multiple regression analysis ($p<0.005$).

https://doi.org/10.1371/journal.pone.0199719.g001

Discussion

We found the preoperative serum level of soluble B7-H4 to be significantly associated with an elevated preoperative peripheral blood neutrophil count in renal cancer patients. These data agreed with our previous report that membrane-bound B7-H4 performed an inhibitory function against neutrophil expansion in a Listeria infection and rheumatoid arthritis model[8, 9]

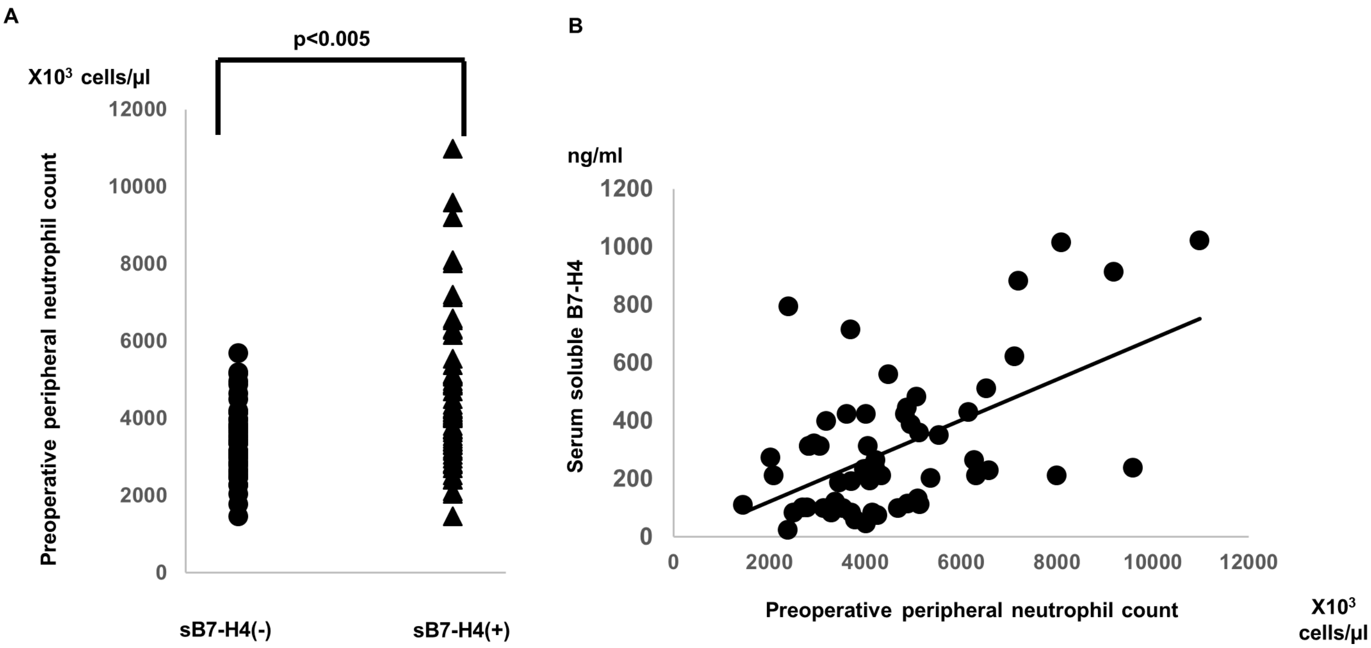

Fig 2. The association between serum soluble B7-H4 and peripheral blood neutrophils. A: The data were analyzed using the Mann-Whitney U test followed by multiple regression analysis ($p<0.005$). **B:** The data summarize the findings of 68 RA patients and were analyzed using Spearman's rank test. $y = 0.07x-19.6$, $R^2 = 0.428$, $p<0.001$.

https://doi.org/10.1371/journal.pone.0199719.g002

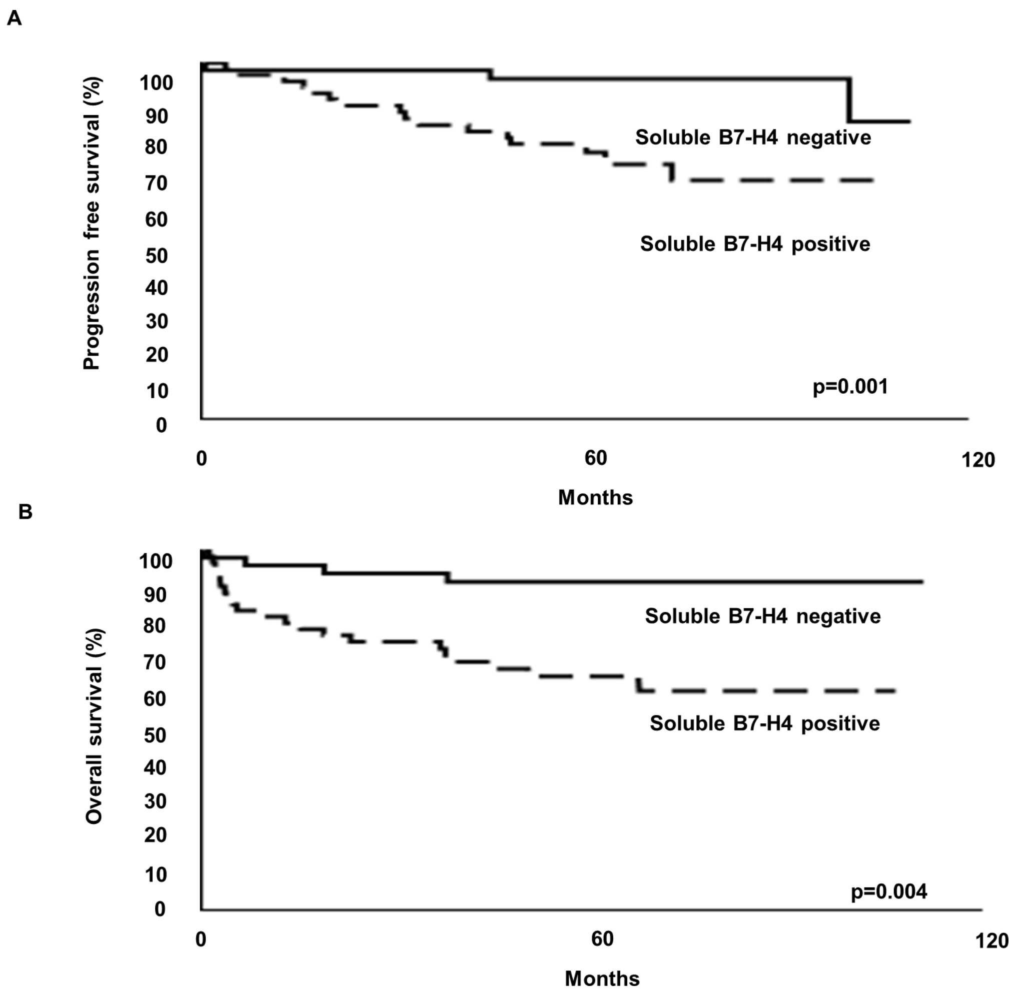

Fig 3. Progression-free survival (A) and overall survival (B) of non-metastatic clear cell renal cell carcinoma patients with and without serum soluble B7-H4.

https://doi.org/10.1371/journal.pone.0199719.g003

Several studies also have reported an association between the overexpression of soluble B7-H4 in the blood of cancer patients and a poor prognosis[10–13]. However, the mechanism of soluble B7-H4 in cancer patients is unclear. Several recent reports have suggested that

Table 2. Univariate and multivariate analyses of risk factors predicting progression free survival in patients with renal cancer.

		Univariate		Multivariate	
Variable	Cutoff	HR (95% CI)	P value	HR (95% CI)	P value
**Age**	≤65 vs >65	-0.102 (-0.508–0.292)	0.613	0.054 (-0.382–0.481)	0.806
**Gender**	Male vs Female	-0.232 (-0.626–0.175)	0.246	-0.336 (-0.761 –0.094)	0.124
**Grade**	G1-2 vs G3-4	-0.499 (-0.954–0.053)	0.039	0.057 (-0.644–0.838)	0.879
**T stage**	T1-2 vs T3-4	-0.316 (-0.747–0.190)	0.171	-0.535 (-1.25–0.201)	0.151
**Preoperative neutrophil count**	≤6000 vs >6000	-0.480 (-0.964–0.136)	0.068	-0.248 (-0.734–0.32)	0.365
**Serum soluble B7-H4**	Negative vs Positive	-0.797 (-1.41 –-0.314)	0.001	-0.949 (-1.59 –-0.316)	0.002

HR hazard ratio, CI confidence interval. Multiple regression model was used for statistical analyses. P value of < 0.05 was considered statistically significant

https://doi.org/10.1371/journal.pone.0199719.t002

Table 3. Univariate and multivariate analyses of risk factors predicting overall survival in patients with renal cancer.

		Univariate		Multivariate	
Variable	Cutoff	HR (95% CI)	P value	HR (95% CI)	P value
**Age**	≤65 vs >65	-0.290 (-0.787–0.167)	0.218	-0.076 (-0.608–0.431)	0.771
**Gender**	Male vs Female	-0.638 (-1.55–0.288)	0.159	-0481 (-0.997–0.024)	0.062
**Grade**	G1-2 vs G3-4	-0.837 (-1.31 –-0.312)	0.0002	-0.311 (-1.07–0.492)	0.442
**T stage**	T1-2 vs T3-4	-0.603 (-1.06 –-0.104)	0.008	-0.625 (-1.39–0.148)	0.112
**Preoperative neutrophil count**	≤6000 vs >6000	-0.501 (-1.06 –-0.231)	0.10	-0.521 (-1.13–0.163)	0.123
**Serum soluble B7-H4**	Negative vs Positive	-0.834 (-1.57 –-0.275)	0.004	-0.868 (-1.72 –-0.177)	0.018

HR hazard ratio, CI confidence interval. Multiple regression model was used for statistical analyses. P value of < 0.05 was considered statistically significant

https://doi.org/10.1371/journal.pone.0199719.t003

neutrophils may also be key players in cancer-associated inflammation[14–16]. Neutrophilia and lymphocytopenia were reported to be predictors of a poor prognosis in some malignancies[17–19]. neutrophil-to-lymphocyte ratio (NLR) is a widely-used marker for systemic inflammatory reactions[20]. A relationship between pretreatment NLR and prognosis has been observed in various malignancies[21–23]. We have also reported that the neutrophil count following IFN-α treatment can serve as a predictive marker for OS in metastatic RCC and that preoperative NLR is an independent predictor of the prognosis of patients with an upper urinary tract urothelial carcinoma[24, 25].

MDSCs are immunosuppressive immature myeloid cells that are elevated in cancer patients and function to suppress T-cell function[26]. In humans, the MDSC has a polymorphonuclear granulocyte morphology[27], suggesting that neutrophilia might account for the increase of MDSCs.

In our previous study, we reported that soluble B7-H4 acts as a decoy molecule to block the inhibitory functions of cell-surface B7-H4[9]. Leung et al. found that cell-surface B7-H4 is able to inhibit protumor MDSCs[28]. MDSCs derived from B7-H4 KO mice suppressed T cell proliferation more potently than their WT counterparts. The elevation of soluble B7-H4 might be able to induce a strong MDCS response. Ohki showed that the peripheral blood MDSC count in patients with gastric and colorectal cancer correlated with the neutrophil count and inversely correlated with the lymphocyte count, indicating a strong correlation with the neutrophil-to-lymphocyte ratio (NLR)[29]. These data suggested that the existence of serum-soluble B7-H4 could be a predictive marker for immunotherapy targeting T lymphocytes. In addition, B7-H4 can be a new target for immunotherapy in renal cancer patients.

Conclusions

The present study showed that the existence of serum-soluble B7-H4 can be an independent predictive marker for PFS and OS in patients with non-metastatic clear cell renal cell carcinoma. To our knowledge, this is the first report of an association between serum soluble B7-H4 and peripheral blood neutrophil count in patients with renal cancer.

Supporting information

S1 Dataset.
(XLSX)

Author Contributions

Conceptualization: Miyuki Azuma, Haruki Kume.

Funding acquisition: Takeshi Azuma, Yujiro Sato.

Investigation: Takeshi Azuma, Yujiro Sato.

Methodology: Tetsukuni Ohno.

Writing – original draft: Takeshi Azuma.

Writing – review & editing: Tetsukuni Ohno, Miyuki Azuma, Haruki Kume.

References

1. Sica GL, Choi IH, Zhu G, Tamada K, Wang SD, Tamura H, et al. B7-H4, a molecule of the B7 family, negatively regulates T cell immunity. Immunity. 2003; 18(6):849–61. PMID: 12818165.
2. Sun Y, Wang Y, Zhao J, Gu M, Giscombe R, Lefvert AK, et al. B7-H3 and B7-H4 expression in non-small-cell lung cancer. Lung Cancer. 2006; 53(2):143–51. https://doi.org/10.1016/j.lungcan.2006.05.012 PMID: 16782226.
3. Awadallah NS, Shroyer KR, Langer DA, Torkko KC, Chen YK, Bentz JS, et al. Detection of B7-H4 and p53 in pancreatic cancer: potential role as a cytological diagnostic adjunct. Pancreas. 2008; 36(2):200–6. https://doi.org/10.1097/MPA.0b013e318150e4e0 PMID: 18376314.
4. Krambeck AE, Thompson RH, Dong H, Lohse CM, Park ES, Kuntz SM, et al. B7-H4 expression in renal cell carcinoma and tumor vasculature: associations with cancer progression and survival. Proc Natl Acad Sci U S A. 2006; 103(27):10391–6. https://doi.org/10.1073/pnas.0600937103 PMID: 16798883; PubMed Central PMCID: PMCPMC1502468.
5. Jiang J, Zhu Y, Wu C, Shen Y, Wei W, Chen L, et al. Tumor expression of B7-H4 predicts poor survival of patients suffering from gastric cancer. Cancer Immunol Immunother. 2010; 59(11):1707–14. https://doi.org/10.1007/s00262-010-0900-7 PMID: 20725832.
6. Mugler KC, Singh M, Tringler B, Torkko KC, Liu W, Papkoff J, et al. B7-h4 expression in a range of breast pathology: correlation with tumor T-cell infiltration. Appl Immunohistochem Mol Morphol. 2007; 15(4):363–70. https://doi.org/10.1097/01.pai.0000213159.79557.71 PMID: 18091377.
7. Yi KH, Chen L. Fine tuning the immune response through B7-H3 and B7-H4. Immunol Rev. 2009; 229 (1):145–51. https://doi.org/10.1111/j.1600-065X.2009.00768.x PMID: 19426220; PubMed Central PMCID: PMCPMC2696224.
8. Zhu G, Augustine MM, Azuma T, Luo L, Yao S, Anand S, et al. B7-H4-deficient mice display augmented neutrophil-mediated innate immunity. Blood. 2009; 113(8):1759–67. https://doi.org/10.1182/blood-2008-01-133223 PMID: 19109567; PubMed Central PMCID: PMCPMC2647680.
9. Azuma T, Zhu G, Xu H, Rietz AC, Drake CG, Matteson EL, et al. Potential role of decoy B7-H4 in the pathogenesis of rheumatoid arthritis: a mouse model informed by clinical data. PLoS Med. 2009; 6(10): e1000166. https://doi.org/10.1371/journal.pmed.1000166 PMID: 19841745; PubMed Central PMCID: PMCPMC2760136.
10. Wang W, Xu C, Wang Y, Yu L, Zhang X. Prognostic values of B7-H4 in non-small cell lung cancer. Bio-markers. 2016:1–16. https://doi.org/10.1080/1354750X.2016.1203997 PMID: 27321138.
11. Zhang C, Li Y, Wang Y. Diagnostic value of serum B7-H4 for hepatocellular carcinoma. J Surg Res. 2015; 197(2):301–6. https://doi.org/10.1016/j.jss.2015.04.034 PMID: 25963168.
12. Dong Q, Ma X. B7-H4 expression is associated with tumor progression and prognosis in patients with osteosarcoma. Biomed Res Int. 2015; 2015:156432. https://doi.org/10.1155/2015/156432 PMID: 25954746; PubMed Central PMCID: PMCPMC4411454.
13. Shi H, Ji M, Wu J, Zhou Q, Li X, Li Z, et al. Serum B7-H4 expression is a significant prognostic indicator for patients with gastric cancer. World J Surg Oncol. 2014; 12:188. https://doi.org/10.1186/1477-7819-12-188 PMID: 24947047; PubMed Central PMCID: PMCPMC4076248.
14. Donskov F, von der Maase H. Impact of immune parameters on long-term survival in metastatic renal cell carcinoma. J Clin Oncol. 2006; 24(13):1997–2005. https://doi.org/10.1200/JCO.2005.03.9594 PMID: 16648500.
15. Ilie M, Hofman V, Ortholan C, Bonnetaud C, Coelle C, Mouroux J, et al. Predictive clinical outcome of the intratumoral CD66b-positive neutrophil-to-CD8-positive T-cell ratio in patients with resectable non-small cell lung cancer. Cancer. 2012; 118(6):1726–37. https://doi.org/10.1002/cncr.26456 PMID: 21953630.
16. Jensen TO, Schmidt H, Moller HJ, Donskov F, Hoyer M, Sjoegren P, et al. Intratumoral neutrophils and plasmacytoid dendritic cells indicate poor prognosis and are associated with pSTAT3 expression in

AJCC stage I/II melanoma. Cancer. 2012; 118(9):2476–85. https://doi.org/10.1002/cncr.26511 PMID: 21953023.

17. Liang S, Sharma A, Peng HH, Robertson G, Dong C. Targeting mutant (V600E) B-Raf in melanoma interrupts immunoediting of leukocyte functions and melanoma extravasation. Cancer Res. 2007; 67 (12):5814–20. https://doi.org/10.1158/0008-5472.CAN-06-4233 PMID: 17575149; PubMed Central PMCID: PMCPMC2724629.

18. Jablonska J, Leschner S, Westphal K, Lienenklaus S, Weiss S. Neutrophils responsive to endogenous IFN-beta regulate tumor angiogenesis and growth in a mouse tumor model. J Clin Invest. 2010; 120 (4):1151–64. https://doi.org/10.1172/JCI37223 PMID: 20237412; PubMed Central PMCID: PMCPMC2846036.

19. Muller I, Munder M, Kropf P, Hansch GM. Polymorphonuclear neutrophils and T lymphocytes: strange bedfellows or brothers in arms? Trends Immunol. 2009; 30(11):522–30. https://doi.org/10.1016/j.it.2009.07.007 PMID: 19775938.

20. Zahorec R. Ratio of neutrophil to lymphocyte counts—rapid and simple parameter of systemic inflammation and stress in critically ill. Bratisl Lek Listy. 2001; 102(1):5–14. PMID: 11723675.

21. Walsh SR, Cook EJ, Goulder F, Justin TA, Keeling NJ. Neutrophil-lymphocyte ratio as a prognostic factor in colorectal cancer. J Surg Oncol. 2005; 91(3):181–4. https://doi.org/10.1002/jso.20329 PMID: 16118772.

22. Yamanaka T, Matsumoto S, Teramukai S, Ishiwata R, Nagai Y, Fukushima M. The baseline ratio of neutrophils to lymphocytes is associated with patient prognosis in advanced gastric cancer. Oncology. 2007; 73(3–4):215–20. https://doi.org/10.1159/000127412 PMID: 18424885.

23. Cho H, Hur HW, Kim SW, Kim SH, Kim JH, Kim YT, et al. Pre-treatment neutrophil to lymphocyte ratio is elevated in epithelial ovarian cancer and predicts survival after treatment. Cancer Immunol Immunother. 2009; 58(1):15–23. https://doi.org/10.1007/s00262-008-0516-3 PMID: 18414853.

24. Azuma T, Matayoshi Y, Nagase Y, Oshi M. Neutrophil number after interferon-alfa treatment is an independent predictive marker of overall survival in metastatic renal cell carcinoma. Clin Genitourin Cancer. 2012; 10(3):180–4. https://doi.org/10.1016/j.clgc.2012.03.006 PMID: 22608529.

25. Azuma T, Matayoshi Y, Odani K, Sato Y, Sato Y, Nagase Y, et al. Preoperative neutrophil-lymphocyte ratio as an independent prognostic marker for patients with upper urinary tract urothelial carcinoma. Clin Genitourin Cancer. 2013; 11(3):337–41. https://doi.org/10.1016/j.clgc.2013.04.003 PMID: 23665132.

26. Ko JS, Bukowski RM, Fincke JH. Myeloid-derived suppressor cells: a novel therapeutic target. Curr Oncol Rep. 2009; 11(2):87–93. PMID: 19216839.

27. Ochoa AC, Zea AH, Hernandez C, Rodriguez PC. Arginase, prostaglandins, and myeloid-derived suppressor cells in renal cell carcinoma. Clin Cancer Res. 2007; 13(2 Pt 2):721s–6s. https://doi.org/10.1158/1078-0432.CCR-06-2197 PMID: 17255300.

28. Leung J, Suh WK. Host B7-H4 regulates antitumor T cell responses through inhibition of myeloid-derived suppressor cells in a 4T1 tumor transplantation model. J Immunol. 2013; 190(12):6651–61. https://doi.org/10.4049/jimmunol.1201242 PMID: 23686485.

29. Ohki S, Shibata M, Gonda K, Machida T, Shimura T, Nakamura I, et al. Circulating myeloid-derived suppressor cells are increased and correlate to immune suppression, inflammation and hypoproteinemia in patients with cancer. Oncol Rep. 2012; 28(2):453–8. https://doi.org/10.3892/or.2012.1812 PMID: 22614133.
